# Mindfulness meditation and bimanual coordination control: study of acute effects and the mediating role of cognition

**DOI:** 10.3389/fpsyg.2023.1162390

**Published:** 2023-05-15

**Authors:** Louise Devillers-Réolon, Jean-Jacques Temprado, Rita Sleimen-Malkoun

**Affiliations:** Aix-Marseille University, CNRS, ISM, Marseille, France

**Keywords:** bimanual coordination, motor control, cognition, acute effects, attention, perceptual inhibition, motor inhibition, brief mindfulness meditation

## Abstract

**Introduction:**

Mindfulness meditation (MM) involves and benefits cognitive functioning, especially attention and inhibition processes, which are also implicated in the control of complex motor skills, such as bimanual coordination. Thus, MM practice could potentially enhance bimanual coordination control through its cognitive benefits. Accordingly, in this study, we investigated the acute effects of a brief MM session on bimanual coordination dynamics, attention, and inhibition abilities, as well as the mediation link between MM’s cognitive and motor improvements.

**Methods:**

Healthy meditation-naïve (novices, *n* = 29) and meditation-experienced participants (meditators, *n* = 26) were randomly assigned to either an active control intervention (attentive listening to a documentary podcast) or a MM intervention (breathing and open monitoring exercise), both lasting 15 min. In the motor domain, pre- and post-tests assessed participants’ ability to intentionally maintain the anti-phase coordination pattern at maximal movement frequency and resist the spontaneous transition to the in-phase pattern. In the cognitive domain, the participants’ attentional, perceptual inhibition and motor inhibition abilities were assessed.

**Results:**

Following both interventions, meditators and novices improved the stability of their anti-phase coordination pattern (*p* = 0.034, *η_p_*^2^ = 0.10) and their attentional performance (*p*’s < 0.001, *η_p_*^2^ > 0.40). Only following the MM intervention, meditators and novices improved their ability to intentionally maintain the anti-phase pattern by delaying or even suppressing the spontaneous transition to in-phase (*p*’s < 0.05, *η_p_*^2^ ≥ 0.11), and improved concomitantly their motor inhibition scores (*p* = 0.011, *η_p_*^2^ = 0.13). No effects were found on perceptual inhibition. The increase in motor inhibition capacities did not however statistically mediate the observed acute effects of MM on bimanual coordination control.

**Conclusion:**

We showed that a single MM session may have acute benefits in the motor domain regardless of the familiarity with MM practice. Although these benefits were concomitant to enhanced attentional and motor inhibition abilities, no formal mediation link could be established between the observed motor and cognitive benefits. This study paves the way for the investigation of the mechanisms underlying MM effects on motor control, as well as longer-term benefits.

## 1. Introduction

Cognitive and motor processes are functionally intertwined, especially with regard to complex movement control (Diamond, 2000; Prinz and Koch, 2009; Leisman et al., 2016; Stuhr et al., 2018). Cognitive interventions are hence progressively gaining interest as a promising way to improve motor function (Chan et al., 2013; Smith-Ray et al., 2015; Slimani et al., 2016). The present study investigated whether mindfulness meditation (MM), which is a mental practice involving attentional regulation (Bishop et al., 2004; Malinowski, 2013) could as well be a suitable candidate to enhance motor control through its cognitive benefits. We tested this hypothesis in a bimanual coordination task, which has been frequently used to study the role of cognition in complex motor skills (Serrien et al., 2007; Temprado et al., 2020).

Mindfulness practice and its benefits have witnessed a soaring scientific interest during the last 15 years (Baminiwatta and Solangaarachchi, 2021). MM can be defined as the training of attention to be fully drawn to the immediate moment with a sense of curiosity, openness, and acceptance (Bishop et al., 2004; Kabat-Zinn, 2017). It invites the meditator to observe their thoughts, feelings, and sensations without any judgment, filter, or expectations. Among the multiple cognitive processes that are mobilized during MM, attention and inhibition play a central role (Bishop et al., 2004; Malinowski, 2013). Attention is necessary to orient, maintain, and supervise the meditator’s focus on the present moment, with or without including a specific object (e.g., focusing on the breath, or openly monitoring anything that occurs in the present experience). Whereas inhibition is necessary to suppress the elaborative processing of thoughts, feelings, sensations and the interferences from irrelevant stimuli (Bishop et al., 2004).

The current literature is supportive of the positive effects of short and long-term MM practice on cognitive functions, such as attention, inhibition, working memory and cognitive flexibility (Chiesa et al., 2011; Gallant, 2016; Cásedas et al., 2019). Even a single brief session of MM, sometimes referred to as mindfulness induction (Creswell, 2017), appears to be beneficial for cognitive performance, especially in tasks involving complex higher-order functions (Gill et al., 2020). Although discrepant results can be found in the literature, a recent study, wherein confounding factors were controlled (familiarity with this practice, individual-dependent response, engagement in the intervention), showed that a single brief MM session (10 min) acutely enhances cognitive performance without the need of any previous practice (Sleimen-Malkoun et al., 2023). These cognitive benefits were observed using the Stroop task (Stroop, 1935) that involves mainly cognitive flexibility, attention, and inhibition. Such finding suggests that MM could be a highly plausible way to improve performance in complex motor tasks involving attentional control and/or inhibition, such as bimanual coordination. However, so far, the effects of MM on motor performance have been scarcely investigated, with a limited interest in the underlying mechanisms of action. The existing studies are mainly limited to the investigation of the effects of long-term MM practice (minimally 4 weeks) on precision control. The authors, who were interested in precision sports (e.g., darts, golf), reported benefits on performance after several weeks of MM practice in elites (John et al., 2011) and in participants with no previous sports practice (Meeûs et al., 2010). The studies that addressed MM effects on motor control using fine motor tasks (tracking or reaching task) showed that 8 week of MM practice improves movement accuracy in meditation-naïve participants (Meeûs et al., 2010; Naranjo and Schmidt, 2012) and experienced meditators (Naranjo and Schmidt, 2012), on the detriment of movement speed (Naranjo and Schmidt, 2012). Some authors (Naranjo and Schmidt, 2012) suggested that MM enhance motor control performance by improving the monitoring of task-relevant perceptual and motor cues, which in return improves the control of movement trajectory. Hence, they hypothesized that MM benefits on motor control could be linked to its effects on cognitive regulation. However, researchers have not yet formally addressed or demonstrated the link between MM effects on motor control and its known cognitive benefits. Furthermore, the effects of short-term MM practice on motor control, which could clarify the underlying mechanisms of action, remain unknown.

To fill the remaining gaps in the literature, the present study investigated the acute effects of a single MM session on motor control and examined their potential link with its cognitive effects on attention and inhibition capacities. To do so, we used a cyclic bimanual coordination task, which is a recognized paradigm to study motor control and the involvement of cognitive resources therein, notably attention (Temprado et al., 1999, 2002) and inhibition (Temprado et al., 2020).

Two stable preferred movement patterns characterize the spontaneous cyclic bimanual coordination: the in-phase and the anti-phase patterns (Kelso, 1984). The in-phase pattern results from the simultaneous activation of homologous muscle groups, giving rise to mirror symmetrical movements with respect to the body midline (e.g., simultaneous pronation and supination of the forearms). Whereas the anti-phase pattern results from simultaneous activation of non-homologous muscle groups, so that one limb moves toward the body midline while the other one moves away from it (e.g., one forearm pronating while the other is supinating). Coordination patterns are characterized by the relative phase (RP) representing the spatiotemporal relationship between the two limbs (0° for the in-phase, 180° for the antiphase) (Haken et al., 1985). Pattern stability, indexed by the magnitude of fluctuations of the relative phase (SDRP), has been shown to be higher for in-phase than for anti-phase coordination pattern (Monno et al., 2002). As a result of this stability difference, when movement frequency increases beyond a given critical value, so-called the transition frequency, a spontaneous and abrupt transition from anti-phase to in-phase occurs (Kelso, 1984; Temprado et al., 2010). This spontaneous dynamic can, however, be modulated by cognitive processes (Schöner and Kelso, 1988; Scholz and Kelso, 1990; Temprado et al., 1999; De Luca et al., 2010). For instance, it has been shown that attention (Temprado et al., 1999; Monno et al., 2000) and inhibition (Temprado et al., 2020) may contribute to delay (Temprado et al., 2002) or, even, to suppress the spontaneous transition from anti-phase to in-phase (Lee et al., 1996). Therefore, we can expect that a session of MM could acutely enhance anti-phase maintenance at critically high movement frequency through its benefits on attention and/or inhibition.

Traditionally, the involvement of attentional processes in bimanual coordination has been assessed *via* the dual-task paradigm that consists in performing simultaneously a bimanual coordination pattern and a reaction time task (Temprado et al., 1999; Monno et al., 2000). Nevertheless, the simultaneous performance of both tasks can create artifact perturbations in motor behavior (Temprado et al., 1999, 2001). To avoid such interferences that can bias pattern stability analysis, we chose to evaluate MM acute effects on attention through a dedicated test.

Regarding inhibition, as bimanual coordination is governed by both perceptual (e.g., visual perception of inter-limb phase relationship) and motor processes (e.g., control of homologous or non-homologous muscles) (Temprado et al., 2003; Bingham, 2004; Carson, 2004; Salter et al., 2004), we adopted the distinction between motor inhibition and perceptual inhibition (Nassauer and Halperin, 2003; Dillon and Pizzagalli, 2007; Lustig et al., 2007). Specifically, perceptual inhibition is defined as the capacity to maintain the focus of attention by preventing interference from task-irrelevant stimuli. Conversely, motor inhibition is considered as the capacity to suppress prevalent, but inappropriate, motor responses. Although this distinction is not common in MM literature, it has been successfully used to investigate the link between cognition and motor control through the study of balance (Redfern et al., 2009, 2018, 2019; Mendelson et al., 2010), and bimanual coordination (Torre et al., in press).

In summary, the overarching objective of this study was to investigate the acute effects of a short MM intervention on motor control and their link with the potential concomitant benefits on attention and inhibition capacities. To this end, we allocated participants with and without previous experience in MM practice (respectively called meditators and novices) to either a MM intervention or an active control intervention (attentive listening). We assessed their engagement in the interventions through dedicated questionnaires. To investigate the effects of the interventions on bimanual coordination control, we tested whether they acutely improved anti-phase coordination pattern stability and maintenance at high movement frequency relative to baseline. We also investigated the interventions’ effects on selective attention, and on perceptual and motor inhibition capacities. Finally, we tested if the observed MM-related cognitive effects statistically mediated the observed motor benefits. Due to the implication of attentional resources in both interventions, better anti-phase stabilization (i.e., lower SDRP) and higher attentional abilities were expected after both interventions, but more so after the MM one. Also, we expected to observe a better ability to maintain the anti-phase pattern (i.e., more trials without transition and more movement cycles before the transition) and better motor and perceptual inhibition abilities only after the MM intervention. All these expected benefits were hypothesized to be independent of previous MM experience. Finally, we hypothesized that MM-specific benefits in the cognitive domain would mediate those observed in the motor domain.

## 2. Materials and methods

### 2.1. Study design and randomization

A two-arm randomized controlled design was used to assess cognitive effects of a 15-min guided MM session compared to a 15-min control intervention requiring attentive listening to a podcast, in meditators and in participants with no prior MM practice (called novices). The randomization was conducted using a computer-generated allocation sequence (MATLAB R2018b, MathWorks, Natick, MA, USA) with a ratio of 1.1. to the experimental intervention (MM) and active control intervention (podcast). It was not stratified but was done through blocks of 5 participants for the meditators and novices to balance enrollment across interventions during the recruitment period.

**Figure 1:**
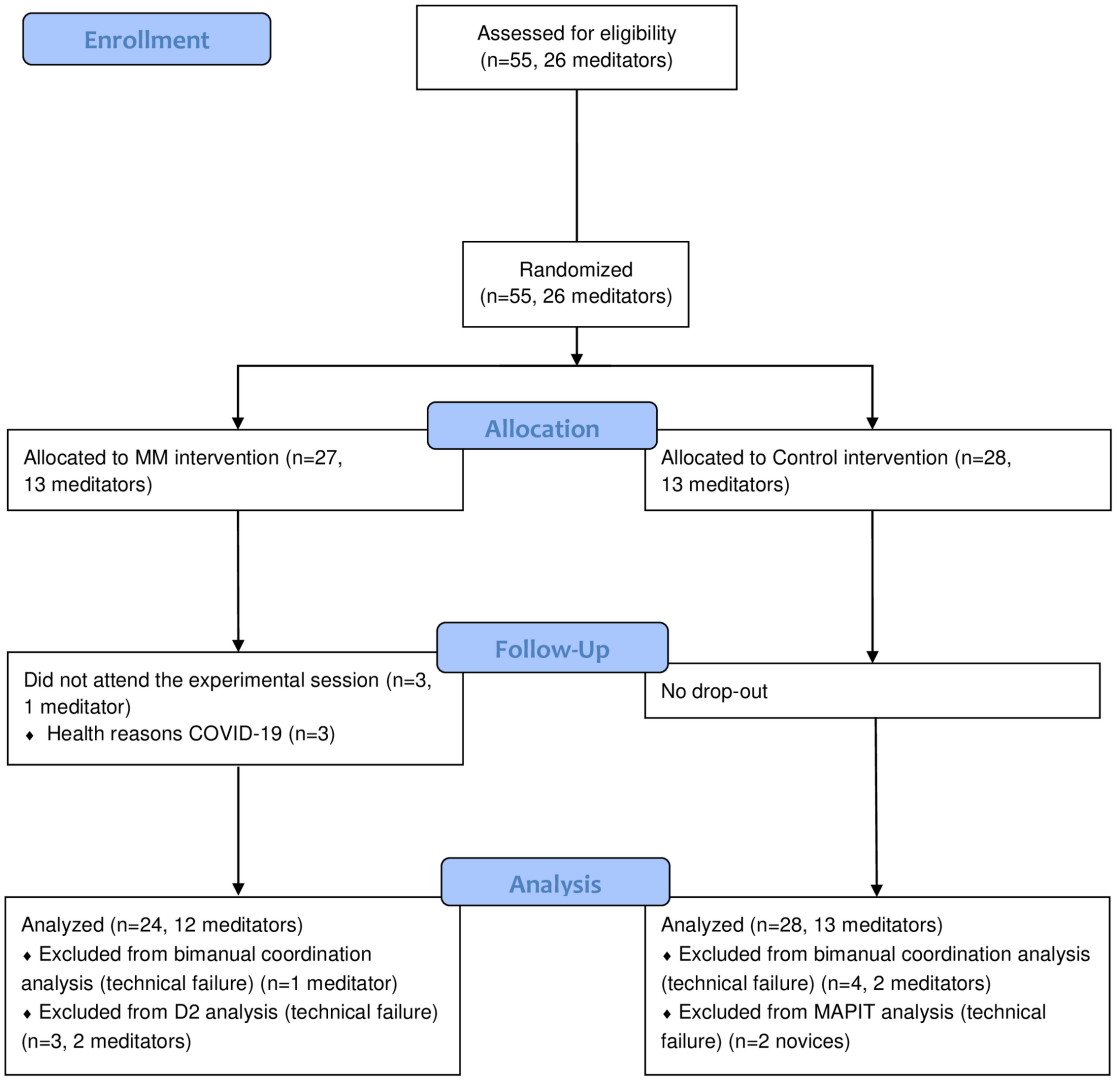
Study design.

Due to the nature of the study, researchers and participants discovered group allocation during the experimental session. Participants were however not informed about the specific objectives of the study. After allocation, the participants were individually tested at baseline (T0) and after their respective 15-min intervention (T1). A 10-min delay post-intervention was respected before testing (T1) in order to optimize the chances of observing acute effects (Luu and Hall, 2017). During this break, the participant completed a short questionnaire to assess their engagement in the allocated intervention (TMS following the MM session, Control Quiz following the control intervention). At T0 and T1, they underwent a bimanual coordination task, an attention test and an inhibition test in a randomized order that was counter-balanced across groups. A summary of the study design can be found in Figure 1. During all the experimental session, the participant was seated in a height adjustable chair without armrests, with their back straight leaning against the seatback.

For sample size calculation we used G*Power3 Software (Faul et al., 2007). It indicated that for a repeated measure analysis of variance (4 groups, 2 tests) at least a total of 48 participants would be required to obtain a large effect size (*f* = 0.45, α = 0.05, 1-β = 0.08, ρ = 0.5). Fifty-five participants were enrolled to compensate any drop-out or problems with data acquisition.

### 2.2. Participants

Fifty-five healthy volunteers were recruited through advertisements on social media platforms and emails, and *via* instructors of MM. Inclusion and exclusion criteria, as well previous MM practice, were verified through a self-reported questionnaire. Inclusion criteria included being aged between 20 and 55 years, being native French speaker or bilingual, and having at least completed a high-school education level. Additionally, for meditators, they needed to report practicing MM at least once a week since minimally 2 months. Exclusion criteria included previous or current neurological or psychiatric disorders, dyslexia, recent or current musculoskeletal problems of the upper limbs, ongoing psychopharmacological medication, uncorrected vision, addiction to drugs or alcoholism, expert sport practice, and for novices, any previous or ongoing practice of any discipline with a mindfulness component (e.g., Yoga, Taï-Chi). Among the 55 recruited participants, 26 were meditators (40.50 ± 10.77 years, 81% women) and 29 had no prior experience in MM practice, which we called novices (32.34 ± 11.99 years, 59% women). The meditators reported practicing MM for at least one time a week (mean = 6.38 sessions per week, range: 1–28 sessions), since minimally 2 months (mean = 48.77 months, range: 2–120 months).

**Figure 2:**
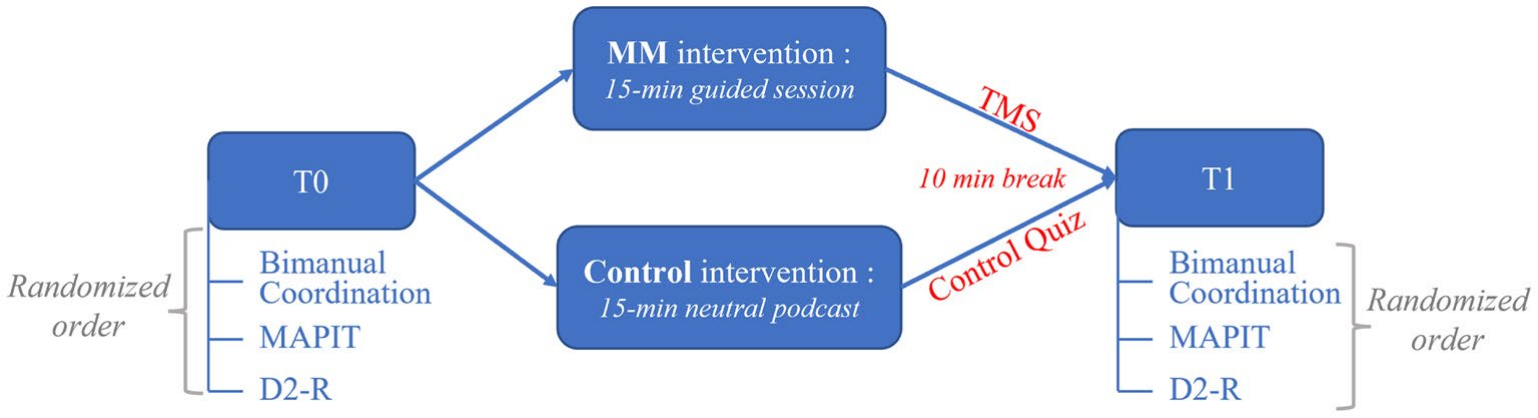
CONSORT flow diagram.

After the randomization procedure, 15 Novices and 13 meditators were allocated to the control intervention (*n* = 28), while 14 novices and 13 meditators were allocated to the MM intervention (*n* = 27). Three participants of those allocated to the MM intervention (2 novices and 1 meditator) abandoned before having completed any test for health reasons (COVID-19). Hence, in the final analyzed sample (*N* = 52), 28 participants were assigned to the control intervention (36.21 ± 11.33 years, 13 meditators, 71% women) and 24 to the MM intervention (37.58 ± 12.68 years, 12 meditators, 66% women). The Consolidated Standards of Reporting Trials (CONSORT) flow diagram of the study can be found in Figure 2.

Demographic characteristics of the four subgroups (novices control, meditators control, novices MM, meditators MM) are presented in Table 1. These sub-groups were not statistically different regarding age (one factor ANOVA: *F*(1,48) = 2.404, *p* = 0.079), and gender (chi-square test: *χ*^2^(3,52) = 2.901, *p* = 0.407).

**Table 1:** Demographic characteristics of the 4 subgroups.

	Control group		MM group	
	Novices	Meditators	Novices	Meditators
Number of participants	15	13	12	12
Age in years (M ± SD)	31.77 ± 11.39	39.31 ± 9.31	32.25 ± 11.37	42.92 ± 12.03
Percentage of women	9	11	7	9
Number of months of MM experience (mean, min-max)	N.A.	53.62, 2–120	N.A.	46.08, 3–120
Number of MM sessions per week (mean, min-max)	N.A.	6.08, 1–28	N.A.	6.08, 1–21

Prior to their enrolment, all participants were given written information about the study, without stating its precise objective or the underlying hypotheses. To limit the contribution of external factors, participants were requested, to abstain from practicing physical activity and MM, drinking alcohol or energy drinks, and ingesting a copious meal for at least 3 h before the experimental session. All participants declared having complied with these requirements.

All participants gave their written informed consent to the experimental procedure that agreed with the Declaration of Helsinki and was approved by the Ethics Committee for Research in Science and Techniques of Physical and Sports Activities (CER STAPS n° IRB00012476-2021-16-11-135).

### 2.3. Interventions

During both interventions, an audio recording was played through a speaker (Beats Pill 2.0 by Dr. Dre, Santa Monica, United States), with the volume adjusted to the participants’ convenience. Both recordings lasted 15 min and featured the same voice with the same (slow) pace of speech. Before the start of the intervention, the participants were invited to adopt a comfortable posture and to close their eyes when ready.

The MM recording was provided by a certified Mindfulness Based Stress Reduction (MBSR) instructor, member of the French organization for the development of mindfulness (ADM). It was a guided fundamental breathing exercise based on classic instructions used in MBSR programs, followed by an open monitoring exercise. The participant was asked to listen attentively and follow, as closely as possible, the given instructions. First, they were instructed to focus their attention on their breathing by counting the number of breath and restart if distracted. This exercise aimed at training the participant to avoid distractions and redirect their awareness to the present moment in a non-reactive and non-elaborative manner (Bishop et al., 2004; Malinowski, 2013; Lutz et al., 2015; Lymeus et al., 2018; Zanesco et al., 2018). Then, the participant was invited to extend their attentional focus to their environment (sensations, background noises, emotions) by mentally putting a word on what they were noticing (for example “noise” when hearing something). Finally, they were asked to bring back their attention to their breath before being invited to open their eyes at the end of the session.

The control recording was a reading in French of a Natural Killers documentary about the Siberian tiger (“Dans l’ombre du tigre,” International Masters Publishers BV 2005). The participant was instructed to listen attentively, not to fall asleep, and not to meditate. This intervention was used to control for the attentional investment that is non-specific to MM.

### 2.4. Measures

#### 2.4.1. Questionnaires

##### 2.4.1.1. Mindfulness state during the MM intervention: toronto mindfulness scale

Following the MM session, participants completed the Toronto Mindfulness Scale (TMS, Lau et al., 2006). This 13-item self-reported questionnaire evaluates the mindful state reached by the participants during the MM session. Six items assess curiosity that is awareness of one’s experience with a quality of curiosity (e.g., *“I experienced myself as separate from my changing thoughts and feelings”*), and seven items assess decentering that is awareness of one’s experience with some distance and disidentification (e.g., *“I experienced myself as separate from my changing thoughts and feelings”*). Participants responded on a 5-point Likert scale from 1 (“not at all”) to 5 (“very much”). A decentering score and a curiosity score are derived by adding up items of each subscale. Higher scores were associated with reaching a more mindful state during the proposed guided-meditation session and hence, to a better auto-reported success in following the instructions during the MM intervention.

In the absence of a scientifically validated French version of this questionnaire, we used a translation/back-translation procedure as described in Brislin (1970) and in Gjersing et al. (2010). The obtained version was tested with 25 respondents (48.24 ± 14.45 years) prior to its final validation and use in this study.

##### 2.4.1.2. Attentiveness during the control intervention: the control quiz

Following the control intervention, participants completed a quiz evaluating how well they attended to the Siberian tiger recording. They had to answer eight multiple-choice questions about information given in the documentary (e.g., *“Where does the Siberian tiger live? Taiga / Steppe / Tundra”*). We derived a score out of 8 by adding up one point for each correct answer. Higher scores were associated to a higher involvement in the control intervention.

#### 2.4.2. Bimanual coordination task

The experimental setup was the same as the one used in Temprado et al. (2010, 2020). It consisted of pronation-supination movements of the forearms in the frontal plane using rotating handles with elbows flexed at 90°. Movements were recorded at a sampling frequency of 100 Hz using potentiometers placed on the axis of rotation of each handle. The participant was instructed to produce the required bimanual movement pattern as accurately and continuously as possible, with a large amplitude (at least 45° around the central position), and in synchrony with an auditory metronome prescribing the required frequency. A full movement cycle had to be performed between two subsequent metronome beats. Fifty cycles of IP and anti-phase bimanual movements at 1 Hz and 1.5 Hz were performed as a familiarization.

Before the formal testing, the participant performed a block of six trials to identify their transition frequency. During these six trials, the metronome frequency was increased stepwise by 0.5 Hz every 10 s, starting from 1 Hz until the occurrence of the transition. The participant was instructed to adopt the AP pattern, and not to intervene if at some point they felt the need to switch to the in-phase pattern. The individual transition frequency was defined as the higher frequency of the six trials at which the participant switched from anti-phase to in-phase.

At T0 and T1, the participant performed six trials of 50 movement cycles at their previously identified transition frequency. They were instructed to maintain the anti-phase pattern as long as possible and resist the spontaneous tendency to switch to the in-phase pattern. If switching occurred, they were asked to continue until the end of the trial with the new adopted in-phase pattern and not to attempt to go back to the initial anti-phase pattern.

For each trial, we calculated the mean RP (in degrees) between the oscillations of the right and left hands, with the right hand as reference. The RP represented the difference in phase angles between the two hands:


$$RP=\frac{t1-t2}{T}\times 360{}^{\circ}$$

with t1 being the time in seconds of the right hand’s peak (maximal or minimal), t2 the time in seconds of the left hand’s peak, and T the period in seconds of the right hand. The onset of the transition was set as the first value of three consecutive cycles of the RP under 135°. The stability and maintenance of the anti-phase pattern was assessed using three complementary indicators: (i) the conventional SDRP (in degrees), (ii) the number of trials without transition (NT) and, (iii) the number of cycles before transition (NC). If there was a transition, RP, SDRP and NC were calculated only before the transition onset. Lower SDRP indicated a higher stability of the anti-phase pattern. Higher NT and NC indicated a better capacity to maintain the anti-phase pattern and to resist the spontaneous transition from anti-phase to in-phase.

#### 2.4.3. Attention test: D2-R

Selective attention was assessed with the D2 test (Brickenkamp, 1962). It is a cancelation task that consists in crossing out targets (the letter “d” with two dashes below and/or above it) while ignoring irrelevant distractors (the letter “d” with one or three dashes, the letter “*p*” with one, two, or three dashes under and/or below it). In this study, we used the D2-R (Brickenkamp et al., 2016), a computerized version implemented in French (Hogrefe Testsystem, HTS 5, Hogrefe Editions). Its internal consistency was shown for a European population of 18–55 years old, as well as its good test–retest reliability (Brickenkamp et al., 2015). At T0 and T1, participants completed 14 successive series including six lines of 10 characters. In each series, participants had to cross as many targets as possible in 20 s while scanning row by row from left to right. For each series, three main scores were calculated: (i) Total Number (TN) of targets scanned at the final cancelation reflecting the rate of processing, (ii) Concentration Capacities (CC) calculated as CC=TN−EO−EC , with EO the number of errors of omission (targets not canceled), and EC the number of errors of confusion (distractors canceled), and (iii) accuracy (E%) which corresponds to the percentage of errors:


$$E\%=\left(\frac{EO+EC}{TN}\right)\times 100.$$

The individual scores were summed across the 13 series (systematically excluding the first one usually related to an adjustment period) and standardized to *T*-scores relative to the European age-specific population. With this quotation, scores are comprised between 25 and 85, with the mean at 50. For a *T*-score of 50, 50% of Europeans of the same age present a superior score and 50% an inferior one. Higher scores were associated with higher selective attention capacities.

#### 2.4.4. Motor and perceptual inhibition test

**Figure 3:**
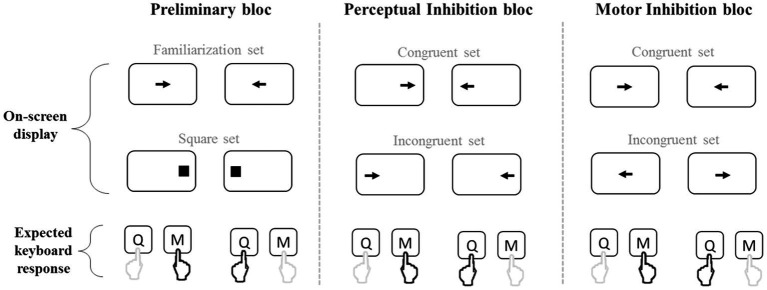
Illustration of the MAPIT. The on-screen display and the expected correct response are presented for all the 12 conditions of the test. Each of the three blocks (Preliminary, Perceptual inhibition, and Motor inhibition) included two sets with two randomized conditions. Only the sets of the perceptual inhibition bloc were intermixed. The correct keyboard letter to press as soon as the on-screen stimulus is presented is indicated by the black pointing hand on the last row (Q with left index or M with right index). For sake of simplicity, in the different blocs, we depicted the conditions requiring the same response in the same column.

Motor and perceptual inhibition abilities were assessed with the MAPIT (Jennings and Mendelson, 2011), that was validated by Nassauer and Halperin (2003). It was programmed with ICE ® software.^1^ The MAPIT is a reaction time task that requires responding as fast as possible according to the direction or the location of a black arrow presented on a white background computer screen (Dell24 P2418HT, 23.8 inches). The displayed arrow can point either left or right, and be positioned at the left, the center, or the right of the screen. The participant responded using a modified AZERTY keyboard on which only two letter keys were kept, with the “Q” key corresponding to the answer “left” and the “M” key corresponding to the answer “right.” They were instructed to press the “Q” key with their left index and “M” key with their right index. A central fixation cross was presented on the screen and disappeared with onset of the first stimulus, which remained on the screen until the participant responded by pressing one of the keys. The test included three blocks of 80 trials each presented in a fixed order (Figure 3): a preliminary block, a perceptual inhibition block, and a motor inhibition block.

The preliminary block included two separated sets (40 trials each, with 20 arrows in each direction). The first one was a familiarization set in which arrows, pointing randomly right, or left (20 trials of each), were presented in the center of the screen. Participants had to press the key corresponding to the direction of the arrow (e.g., press Q with the left finger when the arrow points to the left). The second set was used to elicit and reinforce the prepotent spatial responses. It consisted of 40 black squares randomly presented on the right or the left side (20 trials of each) of the screen. The participant had to respond according to the square’s location (e.g., press Q with the left finger when the square is on the left side of the screen).

The perceptual inhibition block included two sets (40 trials each, with 20 arrows in each direction) that were combined. This resulted in 80 trials randomly interspersed. In the perceptual congruent set the spatial location of the arrow coincided with its pointing direction (e.g., left pointing arrow on the left side of the screen), while in the perceptual incongruent set the spatial location of the arrow conflicted with its pointing direction (e.g., left pointing arrow on the right side of the screen). The participant was required to press the key corresponding to the arrow direction regardless of its location. Hence, in the incongruent trials, the participant had to inhibit the natural tendency of processing the arrow’s location, and instead to focus on its pointing direction.

The motor inhibition block included two separated sets (40 trials each, with 20 arrows in each direction) wherein the arrows were always located at the center of the screen. In the motor congruent set, the participant had to press the key in accordance with the arrows’ direction, while in the incongruent set they were required to press the key opposite to the arrow direction (e.g., press Q with the left finger when the arrow points to the right). Hence, in the incongruent trials the over-learned response that is spatially compatible with the stimulus had to be inhibited.

For each set, the median response time (RT, in milliseconds) of correct trials was calculated. We chose medians instead of means to minimize the influence of possible outliers. The perceptual inhibition (PI) and the motor inhibition (MI) scores were derived from these latter:


$$PI=Median\;RT_{Perceptualincongruent}-Median\;RT_{Perceptualcongruent}$$

$$MI=Median\;RT_{Motorincongruent}-Median\;RT_{Motorcongruent}$$

Lower MI and PI scores were, respectively, associated to higher motor inhibition and perceptual inhibition capacities (Nassauer and Halperin, 2003; Jennings and Mendelson, 2011).

### 2.5. Data processing

Data were processed with MATLAB R2018b (MathWorks, Natick, MA, United States) and Microsoft Excel 2010 (Microsoft Corporation, Impressa systems, Santa Rosa, California, United States).

For the bimanual coordination task, we removed the three first seconds of each trial to ensure the stabilization of the participant’s behavior. Raw data were filtered with a dual-path Butterworth filter (cut-off frequency 10 Hz, order 2), with application of the correction factor (Robertson and Dowling, 2003). Frequency artifacts of the non-sinusoidal signals were removed with an amplitude centering procedure. Trials were discarded from RP, SDRP, and NC computation if a transition appeared before having at least four movement cycles (representing less than 7% of trials).

For the MAPIT, we ensured that all participants had more than 75% of correct answers and that none of the analyzed RT values exceeded 3 s or were below 200 milliseconds.

### 2.6. Data analysis

We statistically analyzed our data using STATISTICA (version 13.3, StatSoft Inc., Tulsa, OK, United States) and JASP (version 0.14.1, JASP Team, Amsterdam, Netherlands).

We report the results of the studied variables in the bimanual coordination task (SDRP, NT, NC), the D2-R (TN, CC, E%) and the MAPIT (MI, PI) as means with within-subject correlation-adjusted error bars (M ± CI), with CI representing the 95% confidence interval normalized to account for the within-subjects design (Cousineau, 2005; Cousineau et al., 2021). Questionnaires’ data (Control Quiz and TMS scores) are reported as means with standard deviation (M ± SD).

Were first compared novices and meditators on questionnaires’ data. For the TMS scores, we used unpaired t-tests after a normality check with Shapiro–Wilk test. For the control questionnaire scores, due to normality violation, we used the U-Mann Whitney test. Then, to investigate the effects of interventions on bimanual coordination and cognitive abilities in meditators and novices, we used a three-ways repeated measures analysis of variance (ANOVA) with test (2: T0, T1) × experience (2: novices, meditators) × intervention (2: MM, control). The level of significance was set to 5% (*p* < 0.05). Effect sizes are reported as Cohen’s d effect size for independent sample t-tests and U-Mann Whitney tests, and partial eta square (*η_p_*^2^) for ANOVAs. When main effects or interactions were statistically significant, Fisher LSD post-hoc comparisons were conducted to test for significant pairwise differences. Finally, mediation analyses were conducted with a bootstrapping procedure (bootstrapped sample, 1,000). This was done to investigate whether MM effects on bimanual coordination were indirect and mediated by its effects on cognitive abilities. We used only motor and cognitive variables significantly impacted by the MM intervention, i.e., for which the ANOVA revealed a significant test × intervention interaction. The predictor was the intervention dichotomously coded as control or MM, the moderator was the T0-T1 change score for the D2-R or MAPIT variables, and the outcome was the T0-T1 change score for bimanual coordination variables. Estimate (*E*), standard error (*SE*) and *value of p* of total direct and indirect effects are reported. For the sake of brevity, only values of statistically significant effects are reported in the text and the figures of the Results section.

Internal consistency of the TMS scores was analyzed using McDonald’s omegas (Dunn et al., 2014).

Due to technical problems some data were corrupted or missing. Specifically, in the bimanual task, data of two novices and two meditators who followed the control intervention, and one meditator who followed the MM intervention were not correctly acquired, and thus could not be analyzed. For the D2, data of one novice and two meditators of the MM group could not be analyzed. Finally, for the MAPIT, data of two novices who followed the control intervention could not be analyzed.

## 3. Results

### 3.1. Questionnaires

The TMS showed good internal consistency (McDonald’s omega) for the curiosity and decentering scores (respectively, 0.70 and 0.88). The mean curiosity and decentering scores of all participants were, respectively, 14.79 ± 5.38 /24 and 19.63 ± 5.02 /28. The unpaired t-tests revealed a significant effect between the meditators and novices only for the decentering score (*t*(22) = −2.99, *p* = 0.007, *d* = 1.221) that was higher in meditators (22.08 ± 4.06) than in novices (17.17 ± 4.00).

Regarding the control recording quiz, the mean obtained score of all participants was 7.32 ± 0.1 /8, with no statistically significant difference between novices and meditators.

### 3.2. Bimanual coordination

**Figure 4:**
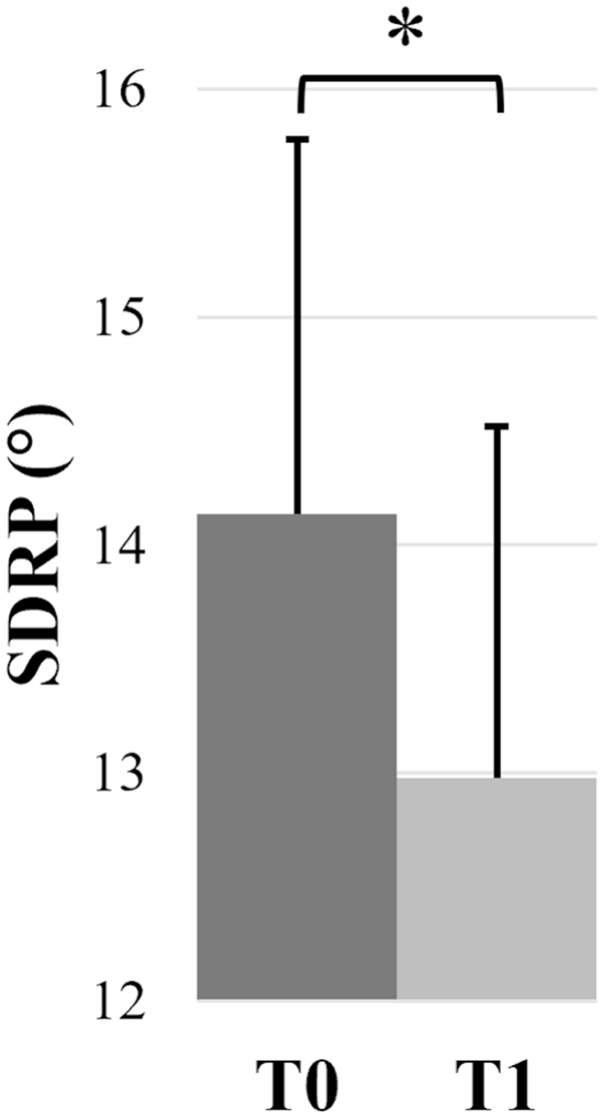
Mean relative phase variability (SDRP) at T0 and T1, irrespective of mindfulness experience and intervention. Error bars represent the normalized 95% confidence interval. *p ≤ 0.05.

**Figure 5:**
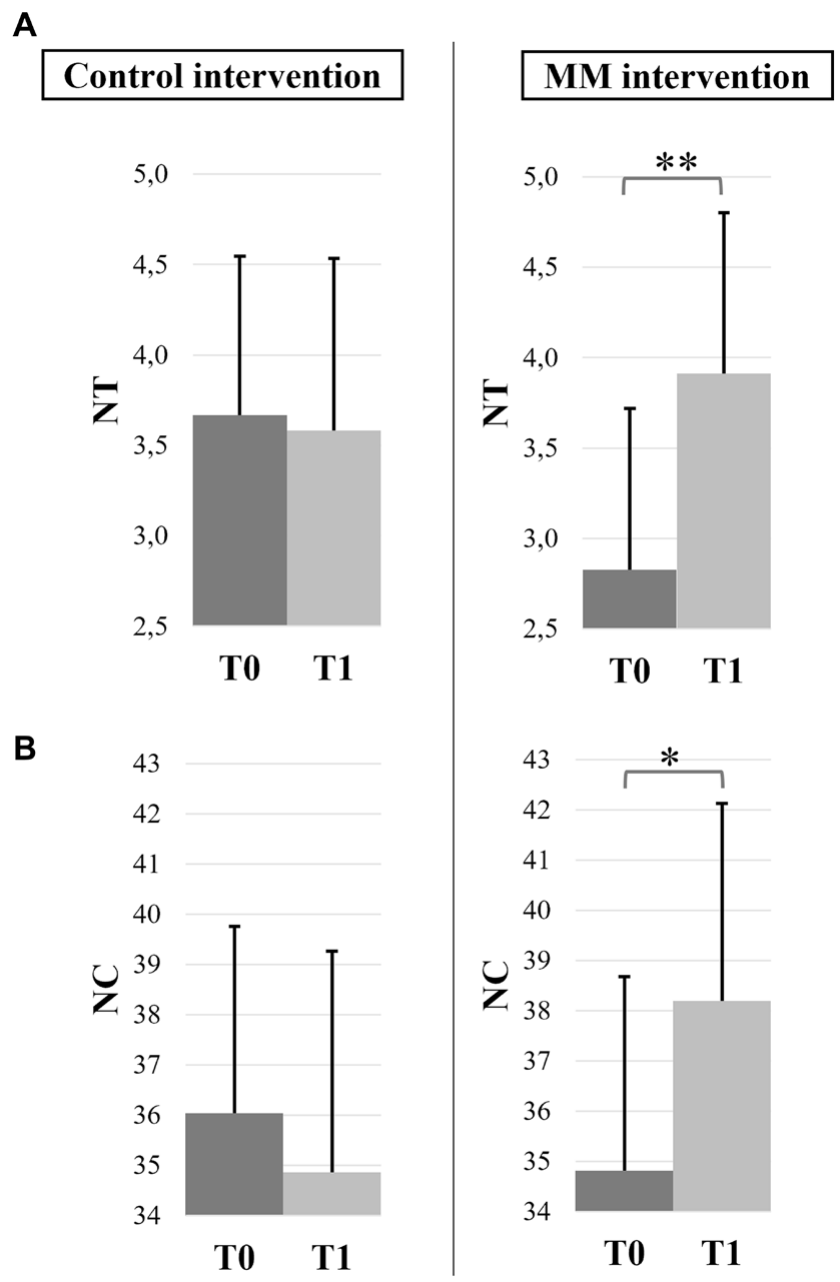
Bimanual coordination performance before (T0) and after (T1) both interventions, irrespective of mindfulness experience. **(A)** Number of trials without transition (NT) and **(B)** Number of cycles before transition (NC). Error bars represent the normalized 95% confidence interval. *p ≤ 0.05 and **p ≤ 0.01.

Mean values and confidence intervals of all bimanual coordination variables of the 4 subgroups are presented in Table 2. For the mean RP, the ANOVA revealed no significant effects, with a grand average close to the required 180° representing the anti-phase pattern (161.37 ± 1.54°). Regarding the SDRP (Figure 4), we observed a significant effect of test (*F*(1,43) = 4.78, *p* = 0.034, *η_p_*^2^ = 0.10), with lower values at T1 (12.98 ± 1.54°) than at T0 (14.14 ± 1.64°). For NT and NC, the ANOVA revealed a significant test × intervention interaction (NT: *F*(1,43) = 7.18, *p* = 0.010, *η_p_*^2^ = 0.14; NC: *F*(1,43) = 5.28, *p* = 0.027, *η_p_*^2^ = 0.11). The post-hoc decompositions showed that more trials without transition (*p* = 0.001) (Figure 5A) and more movement cycles before transition (*p* = 0.018) (Figure 5B) were observed at T1 compared to T0 only after the MM intervention, regardless of previous meditation experience.

**Table 2:** Cognitive and motor performance of the 4 subgroups at T0 and T1.

	Control intervention Mean (CI)				MM intervention Mean (CI)			
	Novices		Meditators		Novices		Meditators	
	T0	T1	T0	T1	T0	T1	T0	T1
Coordination								
RP (°)	161.30 (4.25)	164.03 (3.38)	160.83 (5.51)	159.84 (4.73)	160.89 (4.36)	161.37 (5.24)	160.83 (3.60)	161.42 (4.58)
SDRP (°)	12.86 (2.67)	11.18 (1.98)	14.85 (4.53)	15.20 (4.70)	14.05 (2.64)	13.05 (2.64)	15.03 (3.60)	12.81 (2.69)
NT	3.23 (1.22)	3.23 (1.44)	4.18 (1.26)	4.00 (1.21)	2.92 (0.98)	4.33 (1.17)	2.73 (1.59)	3.46 (1.36)
NC	35.25 (5.17)	33.17 (6.70)	36.98 (5.57)	36.86 (5.55)	36.90 (2.31)	41.67 (2.49)	32.54 (7.65)	34.39 (7.29)
D2								
TN	58.40 (3.15)	63.60 (3.15)	52.00 (4.81)	56.62 (5.15)	56.50 (4.29)	61.50 (4.03)	53.00 (5.48)	57.25 (4.86)
CC	59.27 (2.93)	65.93 (2.97)	52.85 (5.10)	59.62 (2.22)	57.67 (5.00)	63.92 (4.37)	53.33 (5.37)	58.83 (4.98)
E%	51.47 (3.62)	56.40 (2.47)	51.08 (4.73)	58.31 (2.80)	51.75 (4.27)	57.00 (2.71)	49.58 (5.26)	53.58 (4.10)
MAPIT								
PI (ms)	44.07 (19.06)	47.02 (14.65)	44.00 (16.62)	56.87 (19.45)	29.04 (19.21)	36.37 (24.41)	50.03 (15.48)	51.95 (18.39)
MI (ms)	41.48 (18.25)	50.69 (18.62)	104.14 (47.64)	95.82 (41.12)	44.08 (23.86)	22.82 (11.42)	92.31 (23.75)	59.33 (19.03)

### 3.3. Selective attention

**Figure 6:**
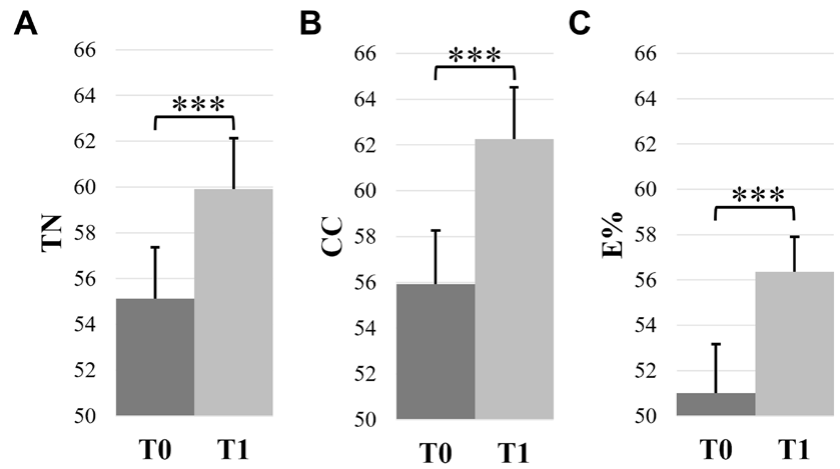
D2 performance at T0 and T1 irrespective of mindfulness experience and intervention. **(A)** total number of targets scanned (TN), **(B)** concentration capacities (CC), and **(C)** accuracy (E%). Error bars represent the normalized 95% confidence interval. ***p ≤ 0.001.

Mean values and confidence intervals of all D2 variables of the 4 subgroups are presented in Table 2. For TN, we found a statistically significant effect of experience (*F*(1,48) = 6.00, *p* = 0.018, *η*_p_^2^ = 0.11) and test (*F*(1,48) = 382.00, *p* < 0.001, *η_p_*^2^ = 0.89). TN was higher in novices (60.11 ± 2.68) than in meditators (54.70 ± 3.59), and higher at T1 (59.90 ± 2.23) than at T0 (55.12 ± 2.25) (Figure 6A). For CC, the ANOVA revealed a statistically significant effect of experience (*F*(1,48) = 5.87, *p* = 0.019, *η_p_*^2^ = 0.11) and test (*F*(1,48) = 114.83, *p* < 0.001, *η_p_*^2^ = 0.71) (Figure 6B). CC was higher in novices (61.79 ± 2.87) than in meditators (56.16 ± 3.74), and higher at T1 (62.25 ± 2.27) than at T0 (55.92 ± 2.34). For E%, the ANOVA revealed only a statistically significant effect of test (*F*(1,48) = 36.58, *p* < 0.001, *η_p_*^2^ = 0.43), with higher score at T1 (53.36 ± 1.54) compared to T0 (51.00 ± 2.17) (Figure 6C).

### 3.4. Motor and perceptual inhibition

**Figure 7:**
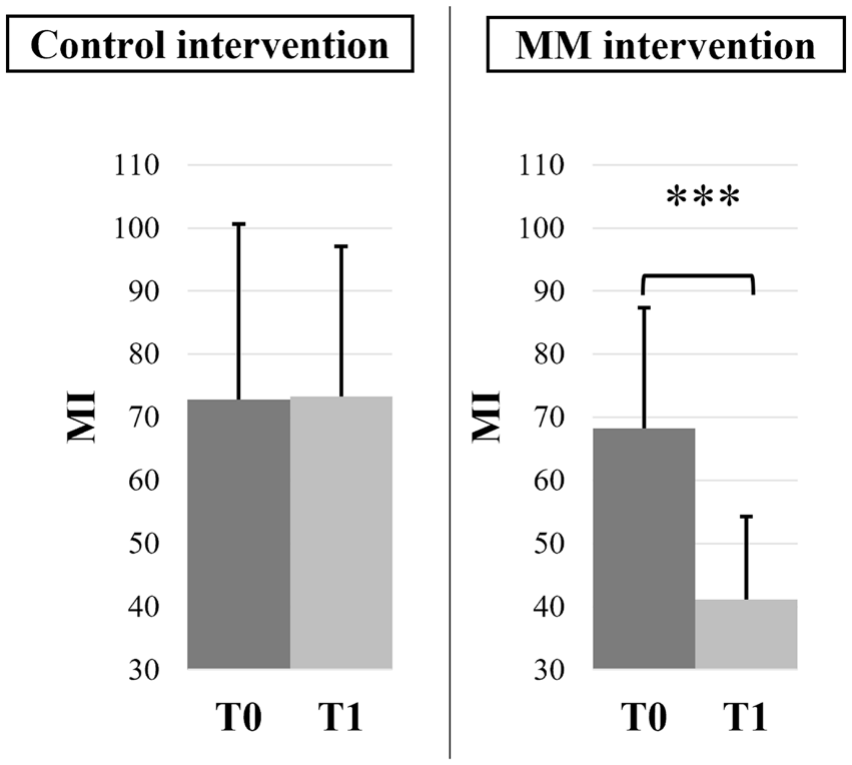
Motor inhibition scores (MI) for both interventions, at T0 and T1 irrespective of mindfulness experience. Error bars represent the normalized 95% confidence interval. ***p ≤ 0.001.

Mean values and confidence intervals of MI and PI scores of the 4 subgroups are presented in Table 2. For the PI score, we found no statistically significant effects. For the MI score, the ANOVA revealed a statistically significant effect of experience (*F*(1,46) = 12.47, *p* < 0.001, *η_p_*^2^ = 0.21) with lower scores in novices, test (*F*(1,46) = 6.59, *p* = 0.014, *η_p_*^2^ = 0.13), and test × intervention interaction (*F*(1,46) = 7.04, *p* = 0.011, *η_p_*^2^ = 0.13). The post-hoc decomposition showed, a reduction of MI at T1 compared to T0 (*p* < 0.001) only after the MM intervention and regardless of past meditation experience (Figure 7). The post-hoc decomposition also showed that at T1, the MI scores of the MM group were significantly lower than those of the control group at T0 (*p* = 0.034) and at T1 (*p* = 0.031).

### 3.5. Mediation analysis

As results showed significant MM effects on motor inhibition performance, we defined the delta MI as the possible mediator of the MM effects on motor performance in the mediation analysis. Results revealed that while the total effect of the intervention on NT was significant (*E* = 1.16, *SE* = 0.42, *p* = 0.006), the indirect effect through delta MI was not statistically significant. Similar results were found for NC with a statistically significant total effect of the intervention (*E* = −0.45, *SE* = 1.87, *p* = 0.016) but no statistically significant indirect effect through delta MI.

## 4. Discussion

The present study investigated the acute effects of a single brief MM session on motor control and their link with concomitant benefits on attention and inhibition. We used a cyclic bimanual coordination task to assess motor control performance, the D2 test to assess selective attention abilities, and the MAPIT to assess motor and perceptual inhibition abilities. We tested participants who were novices or meditators at baseline and following either a 15-min guided MM session or an active control intervention requiring an attentive listening to a 15-min neutral podcast. As a prerequisite, we verified the participants’ engagement in the proposed interventions through dedicated questionnaires.

### 4.1. Participants’ engagement in the interventions

The participants’ TMS scores, which reflect the mindful state reached during the MM session, were similar to those reported in the literature following a similar short MM session (Caldera, 2017). Our decentering scores were even higher [19.63 ± 5.02 in our study and 14.06 ± 5.32 in (Caldera, 2017)]. This is not surprising as participants enrolled in Caldera’s study were novices (Caldera, 2017), while part of ours were meditators. Effectively, our statistical analysis showed higher decentering scores in meditators suggesting that they were more able to decenter themselves from the lived experience than the novices. Regarding the control quiz that was completed following the control intervention, participants showed high scores irrespective of their previous experience in MM. We conclude that, as requested, they carefully listened to the podcast. Overall, the questionnaires’ results suggest that all participants succeeded in following the instructions of their allocated intervention.

### 4.2. Acute effects of MM on bimanual coordination

As both MM and control interventions implicated attentional resources, we were expecting to observe better stabilization of bimanual coordination, reflected by lower inter-limb coordination variability, after both interventions, but more so after MM. Our results showed that both interventions reduced coordination variability, however to a similar extent. Additionally, the MM intervention was expected to enhance bimanual coordination maintenance of the anti-phase pattern by increasing the number of trials without transition and the number of cycles before transition independent of previous MM experience. This was fully confirmed by our results. Our findings lend credence to the hypothesis that intentional maintenance of the anti-phase pattern at high frequency may involve two separate mechanisms, both affected by MM practice: (i) an inhibitory mechanism delaying and suppressing the emergence of the IP pattern as a spontaneous response, and (ii) a pattern stabilization mechanism, presumably resulting from an increase in inter-limb coupling strength (see Temprado et al., 1999, for a detailed development). Moreover, they suggest that the solicitation of attentional resources enhances the pattern stabilization mechanism, while the MM component of orienting attention to the present moment enhances more specifically the inhibitory mechanism (Bishop et al., 2004). The results of the attentional test that we discuss in the following subsection corroborate this assumption.

Regarding the contribution to MM literature, the observed acute benefits of MM practice on bimanual control adds on to those previously reported on precision control in novices following longer interventions (8 weeks, Meeûs et al., 2010; Naranjo and Schmidt, 2012). It extends the actual knowledge by showing that a single 15-min MM session is sufficient to observe transient benefits on motor control independent of whether participants had or had not a previous experience in MM.

### 4.3. Acute effects of MM on selective attention

We were expecting both interventions to enhance selective attention in both meditators and novices, but more so following MM. Our results showed indeed higher scores in the selective attention test after both interventions irrespective of previous MM experience, however to a similar extent for both. These results suggest that the solicitation of attentional resources within or outside MM practice could enhance selective attention, which is the ability to select perceptual task-relevant information. This observation goes hand in hand with the reduction of bimanual coordination variability that was observed after both interventions.

These findings add on to the previously reported benefits of MM on selective attention in novices following longer interventions (8 weeks, Jha et al., 2007; Jensen et al., 2012). The results of this study corroborate those of a recent work (Sleimen-Malkoun et al., 2023) on acute cognitive benefits of MM. That study showed that when controlling possible confounding factors, notably the participant’s engagement in the intervention, a unique MM session can acutely enhance cognitive performance, including selective attention, in both meditators and novices.

### 4.4. Acute effects of MM on motor and perceptual inhibition

We were expecting MM intervention to enhance motor and perceptual inhibition capacities in both meditators and novices. Our results partially confirmed this assumption, as only motor inhibition scores showed a statistically significant improvement following the MM session. This was equally the case for novices and meditators.

Our results suggest that a single MM session improves the ability to keep up with the task goal by suppressing prevalent responses, without the need for any previous experience in MM. However, it does not seem to be enough to help in preventing the consequences of interferences from task-irrelevant perceptual (visual) stimuli. These observed benefits of MM on motor inhibition appear consistent with a previous work on acute effects of MM on habitual responding (Wenk-Sormaz, 2005). That study showed that a single 20-min MM session can enhance the inhibition of prevalent verbal responses. The present study extends previous findings by showing that meditators and novices may equally benefit from MM. We could interpret the reduced automatic responding following MM as a de-automatization of the mental processes that shape and interpret perceptual stimuli (Lutz et al., 2008). It might be related to the development of a present-centered awareness and to the decoupling between the perceived/internal experiences and the overt behavior (Bishop et al., 2004; Levin et al., 2015). This has been reported in several behavioral areas, such as chronic pain or depression (Levin et al., 2015), while our results suggest that it might extend also to cognitive performance.

However, due to methodological heterogeneity, it’s not straightforward to compare our results with the available literature. An original and crucial aspect of the current study is the use of a single validated cognitive test (the MAPIT) to assess the effects of MM on motor and perceptual inhibition. Indeed, so far to our knowledge, the effects of MM practice on perceptual and motor inhibition have been investigated separately through different task paradigms (e.g., Go-Stop, Go/No-go for motor inhibition; Stroop, Hayling task for perceptual inhibition) and protocols, with no comparison between meditators and novices. When using separate tasks based on different frameworks, inconsistent results have been found following short-term MM practice. For perceptual inhibition, some studies reported benefits [e.g., (Luu and Hall, 2017)] while others reported no effects [e.g., (Polak, 2009)]. Regarding motor inhibition, which has been scarcely investigated, studies reported short-term MM effects at the neural level (Andreu et al., 2018), but not at the behavioral level (Andreu et al., 2018; Jaiswal et al., 2020; Baranski, 2021).

### 4.5. Mediation of MM’s benefits on bimanual coordination through Its motor inhibition benefits

Due to the functional link between cognition and motor control (Diamond, 2000; Prinz and Koch, 2009; Leisman et al., 2016; Stuhr et al., 2018), especially during bimanual coordination [e.g., (Temprado et al., 1999, 2020; Monno et al., 2000)], we were expecting that MM benefits on cognition could mediated its observed effects on motor control. Since no statistically significant test × intervention interaction was found for attentional and coordination variability benefits, and since no benefits were found on perceptual inhibition, we did not conduct mediation analysis with these variables. It was only conducted with the delta MI (score change in motor inhibition) as the mediator, and delta NT (change in number of trials without transition) or delta NC (change in number of cycles before transition) as the outcome.

Our results revealed no statistically significant indirect effect of MM on motor variables through delta MI. Hence, despite the fact that the observed MM acute effects on motor control and motor inhibition were concomitant and converging toward reduced reflexive responding, we found no formal evidence for a mediation link between them. Nevertheless, this result does not exclude that MM benefits on cognitive functions could mediated its benefits on motor control at a longer-term, for instance, or through other functions. A plausible explanation of our findings is that the motor inhibition aspect assessed by the MAPIT is not specifically implicated in the intentional maintenance of anti-phase bimanual coordination pattern at transition frequency. This seems to be corroborated by a recent work (Sleimen-Malkoun et al., accepted) reporting that intentional maintenance of the anti-phase pattern is significantly correlated with perceptual inhibition capacities, but not with motor inhibition, as measured by the MAPIT. Moreover, the motor inhibition evaluated in the MAPIT might not be of the exact same nature as the one involved in the bimanual coordination task. Indeed, it has to be noted that the MAPIT implicates a discrete perceptual-motor task, while the bimanual coordination paradigm is a continuous cyclic task belonging to a separate movement class (Hogan and Sternad, 2007). It is noteworthy to acknowledge that motor inhibition processes involved in canceling prepared discrete movements and stopping ongoing rhythmic movements might be different (Hervault et al., 2019, 2021).

### 4.6. Conclusions, limitations and future directions

The present study offers evidence on the efficacy of a short MM session to enhance motor control, as well as selective attention and motor inhibition in healthy adults. These acute improvements were reflected by a better ability to stabilize and maintain intentionally the anti-phase bimanual coordination pattern, as well as a better ability to select perceptual task-relevant information and to suppress dominant responses. Orienting attention to the present moment, which is a specific component of MM, seems to be crucial in the ability to maintain intentionally the anti-phase bimanual coordination pattern and for motor inhibition improvement. Since we tested both meditators and novices and assessed the participants’ engagement in the interventions, one can conclude that these effects were not out of familiarity with mindfulness practice. However, although we found concomitant and coherent positive effects of MM on motor control and motor inhibition, we could not establish a formal mediation link between them. Overall, the demonstrated acute effects support the interest of MM as a training method in both motor and cognitive domains. In addition, it paves the way for the investigation of the mechanisms underlying MM effects on motor control.

These encouraging findings come, however, with some limitations that need to be addressed in future work. In the present protocol, the number of used cognitive tests had to be limited since acute effects are transient and their duration is still unknown. Hence, a first limitation is the difficulty of specifically testing all the relevant cognitive functions that could hold the mediation link. In future research, it would be interesting to assess cognitive flexibility and working memory, knowing their relevance to complex motor skills (Rigoli et al., 2012; Stuhr et al., 2018) and their improvement following MM (Chiesa et al., 2011; Gallant, 2016). Another limitation is the use of only an active control group. As suggested by Davidson and Kazsniak (Davidson and Kaszniak, 2015), a rigorous approach must include several control groups designed to rule out all alternative explanatory mechanisms of the expected effects. Relative to a passive intervention that does not significantly tap into attentional resources, it may have been possible to investigate the link between attentional and motor benefits following the MM intervention. Additionally, we recognize that one cannot exclude the possibility that the increase in D2 scores following both interventions could result, at least in part, from a repetition effect, as previously suggested (Prätzlich et al., 2016). Although the D2’s test–retest reliability has been proven even within 1 h (Brickenkamp et al., 2016), adding a second baseline test would further confirm this reliability. A larger sample size would be also needed to confirm the robustness of the observed benefits. Finally, using neuro-imaging techniques in combination with behavioral assessment would be of great interest to reveal the neurophysiological underpinnings of the studied effects.

## Data availability statement

The raw data supporting the conclusions of this article will be made available by the authors, without undue reservation.

## Ethics statement

The studies involving human participants were reviewed and approved by Ethics Committee for Research in Science and Techniques of Physical and Sports Activities (CER STAPS n° IRB00012476-2021-16-11-135). The patients/participants provided their written informed consent to participate in this study.

## Author contributions

LD-R, J-JT, and RS-M authors participated in conceiving the study design. LD-R conducted the experiment and analyzed the data. All authors contributed to the article and approved the submitted version.

## Conflict of interest

The authors declare that the research was conducted in the absence of any commercial or financial relationships that could be construed as a potential conflict of interest.

## Publisher’s note

All claims expressed in this article are solely those of the authors and do not necessarily represent those of their affiliated organizations, or those of the publisher, the editors and the reviewers. Any product that may be evaluated in this article, or claim that may be made by its manufacturer, is not guaranteed or endorsed by the publisher.

## Footnotes

^1^https://trello.com/b/EtNCNrZH/ice

## References

[ref1] Andreu C. I. Cosmelli D. Slagter H. A. Franken I. H. A. (2018). Effects of a brief mindfulness-meditation intervention on neural measures of response inhibition in cigarette smokers. PLoS One 13, e0191661–e0191616. doi: 10.1371/journal.pone.0191661, PMID: 29370256 PMC5784955

[ref2] Baminiwatta A. Solangaarachchi I. (2021). Trends and developments in mindfulness research over 55 years: a Bibliometric analysis of publications indexed in web of science. Mindfulness 12, 2099–2116. doi: 10.1007/s12671-021-01681-x, PMID: 34306245 PMC8282773

[ref3] Baranski M. F. S. (2021). No state effects of brief mindfulness meditation on the executive functions of inhibition, shifting, and updating. J. Cogn. Enhanc. 5, 311–329. doi: 10.1007/s41465-020-00198-w

[ref4] Bingham G. P. (2004). A perceptually driven dynamical model of bimanual rhythmic movement (and phase perception). Ecol. Psychol. 16, 45–53. doi: 10.1207/s15326969eco1601_6

[ref5] Bishop S. R. Lau M. Shapiro S. Carlson L. Anderson N. D. Carmody J.. (2004). Mindfulness: a proposed operational definition. Clin. Psychol. Sci. Pract. 11, 230–241. doi: 10.1093/clipsy/bph077

[ref6] Brickenkamp R. (1962). Aufmerksamkeits-Belastungs-Test Handanweisung d-2 [The d2 Test of Attention] (1st Edn.). Göttingen: Hogrefe.

[ref7] Brickenkamp R. Schmidt-Atzert L. Liepmann D. (2015). D2-R Version informatisée.

[ref8] Brickenkamp R. Schmidt-Atzert L. Liepmann D. (2016). D2-R: tests d’attention concentrée-révisée: Supplément pour la version innformatisée. France: Hogrefe

[ref9] Brislin R. W. (1970). Back-translation for cross-cultural research. J. Cross-Cult. Psychol. 1, 185–216. doi: 10.1177/135910457000100301

[ref10] Caldera C. A. (2017). Comparing the effects of mindfulness meditation and relaxation in a brief laboratory induction. Theses Diss. 106

[ref11] Carson R. G. (2004). “Governing coordination. Why do muscles matter?” in Coordination dynamics: Issues and trends. eds. Jirsa J. A. S. Kelso V. K. I. (Berlin, Heidelberg: Springer), 141–154.

[ref12] Cásedas L. Pirruccio V. Vadillo M. A. Lupiáñez J. (2019). Does mindfulness meditation training enhance executive control? A systematic review and Meta-analysis of randomized controlled trials in adults. Mindfulness 11, 411–424. doi: 10.1007/s12671-019-01279-4

[ref13] Chan J. S. Y. Yan J. H. Payne V. G. (2013). “Cognitive training enhances motor performance and learning” in Motor behavior and control: New research. eds. Marco L. Fuchs M. (New York: Nova Science Publishers, Inc.), 41–58.

[ref14] Chiesa A. Calati R. Serretti A. (2011). Does mindfulness training improve cognitive abilities? A systematic review of neuropsychological findings. Clin. Psychol. Rev. 31, 449–464. doi: 10.1016/j.cpr.2010.11.003, PMID: 21183265

[ref15] Cousineau D. (2005). Confidence intervals in within-subject designs: a simpler solution to Loftus and Masson’s method. Tutor. Quant. Methods Psychol. 1, 42–45. doi: 10.20982/tqmp.01.1.p042

[ref16] Cousineau D. Goulet M. A. Harding B. (2021). Summary plots with adjusted error bars: the superb framework with an implementation in R. Adv. Methods Pract. Psychol. Sci. 4:251524592110351. doi: 10.1177/25152459211035109

[ref17] Creswell J. D. (2017). Mindfulness interventions. Annu. Rev. Psychol. 68, 491–516. doi: 10.1146/ANNUREV-PSYCH-042716-051139 27687118

[ref18] Davidson R. J. Kaszniak A. W. (2015). Conceptual and methodological issues in research on mindfulness and meditation. Am. Psychol. 70, 581–592. doi: 10.1037/a0039512, PMID: 26436310 PMC4627495

[ref19] De Luca C. Jantzen K. J. Comani S. Bertollo M. Kelso J. A. S. (2010). Striatal activity during intentional switching depends on pattern stability. J. Neurosci. 30, 3167–3174. doi: 10.1523/JNEUROSCI.2673-09.2010, PMID: 20203176 PMC3842453

[ref20] Diamond A. (2000). Close interrelation of motor development and cognitive development and of the cerebellum and prefrontal cortex. Child Develop. 71, 44–56. doi: 10.1111/1467-8624.00117, PMID: 10836557

[ref21] Dillon D. G. Pizzagalli D. A. (2007). Inhibition of action, thought, and emotion: a selective neurobiological review. Appl. Prev. Psychol. 12, 99–114. doi: 10.1016/j.appsy.2007.09.004, PMID: 19050749 PMC2396584

[ref22] Dunn T. J. Baguley T. Brunsden V. (2014). From alpha to omega: a practical solution to the pervasive problem of internal consistency estimation. Br. J. Psychol. 105, 399–412. doi: 10.1111/bjop.12046, PMID: 24844115

[ref23] Faul F. Erdfelder E. Lang A.-G. Buchner A. (2007). G*power 3: a flexible statistical power analysis for the social, behavioral, and biomedical sciences. Behav. Res. Methods 39, 175–191. doi: 10.3758/BF03193146, PMID: 17695343

[ref24] Gallant S. N. (2016). Mindfulness meditation practice and executive functioning: breaking down the benefit. Conscious. Cogn. 40, 116–130. doi: 10.1016/j.concog.2016.01.005, PMID: 26784917

[ref25] Gill L. N. Renault R. Campbell E. Rainville P. Khoury B. (2020). Mindfulness induction and cognition: a systematic review and meta-analysis. Conscious. Cogn. 84:102991. doi: 10.1016/j.concog.2020.102991, PMID: 32739799

[ref26] Gjersing L. Caplehorn J. R. Clausen T. (2010). Cross-cultural adaptation of research instruments: language, setting, time and statistical considerations. BMC Med. Res. Methodol. 10:13. doi: 10.1186/1471-2288-10-13, PMID: 20144247 PMC2831007

[ref27] Haken H. Kelso J. A. S. Bunz H. (1985). A theoretical model of phase transitions in human hand movements. Biol. Cybern. 51, 347–356. doi: 10.1007/BF00336922 3978150

[ref28] Hervault M. Huys R. Buisson J. C. Francheteau M. Siguier P. Zanone P. G. (2021). To start or stop an action depends on which movement we perform: an appraisal of the horse–race model. Acta Psychol. 217:103332. doi: 10.1016/j.actpsy.2021.103332 33991795

[ref29] Hervault M. Huys R. Farrer C. Buisson J. C. Zanone P. G. (2019). Canceling discrete and stopping ongoing rhythmic movements: do they involve the same process of motor inhibition? Hum. Mov. Sci. 64, 296–306. doi: 10.1016/j.humov.2019.02.010, PMID: 30825763

[ref30] Hogan N. Sternad D. (2007). On rhythmic and discrete movements: reflections, definitions and implications for motor control. Exp. Brain Res. 181, 13–30. doi: 10.1007/s00221-007-0899-y, PMID: 17530234

[ref31] Jaiswal S. Tsai S.-Y. Juan C.-H. Liang W.-K. Muggleton N. G. (2020). Exploring the impact of a brief mindfulness induction on motor inhibitory control. Exp. Results 1, 1–8. doi: 10.1017/exp.2020.29

[ref32] Jennings J. Mendelson D. (2011). Detecting age differences in inhibition processes with a test of perceptual and motor inhibition. Exp. Aging Res. 37, 179–197. doi: 10.1080/0361073X.2011.554512.Detecting, PMID: 21424956 PMC3064447

[ref33] Jensen C. G. Vangkilde S. Frokjaer V. Hasselbalch S. G. (2012). Mindfulness training affects attention-or is it attentional effort? J. Exp. Psychol. Gen. 141, 106–123. doi: 10.1037/a0024931, PMID: 21910559

[ref34] Jha A. P. Krompinger J. Baime M. J. (2007). Mindfulness training modifies subsystems of attention. Cogn. Affect. Behav. Neurosci. 7, 109–119. doi: 10.3758/CABN.7.2.109, PMID: 17672382

[ref35] John S. Verma S. K. Khanna G. L. (2011). The effect of mindfulness meditation on HPA-Axis in pre-competition stress in sports performance of elite shooters. Natl. J. Integr. Res. Med. 2, 15–21.

[ref36] Kabat-Zinn J. (2017). Too early to tell: the potential impact and challenges—ethical and otherwise—inherent in the mainstreaming of dharma in an increasingly dystopian world. Mindfulness 8, 1125–1135. doi: 10.1007/s12671-017-0758-2, PMID: 28989546 PMC5605584

[ref37] Kelso J. A. S. (1984). Phase transitions and critical behavior in human bimanual coordination. Am. J. Physiol. Regul. Integr. Comp. Physiol. 246, R1000–R1004. doi: 10.1152/ajpregu.1984.246.6.r1000 6742155

[ref38] Lau M. A. Bishop S. R. Segal Z. V. Buis T. Anderson N. D. Carlson L.. (2006). The Toronto mindfulness scale: development and validation. J. Clin. Psychol. 62, 1445–1467. doi: 10.1002/jclp, PMID: 17019673

[ref39] Lee T. D. Blandin Y. Proteau L. (1996). Effects of task instructions and oscillation frequency on bimanual coordination. Psychol. Res. 59, 100–106. doi: 10.1007/BF01792431, PMID: 8810585

[ref40] Leisman G. Moustafa A. Shafir T. (2016). Thinking, walking, talking: Integratory motor and cognitive brain function. Front. Public Heal. 4:94. doi: 10.3389/fpubh.2016.00094, PMID: 27252937 PMC4879139

[ref41] Levin M. E. Luoma J. B. Psychotherapy--clinic P. Haeger J. (2015). Decoupling as a mechanism of change in mindfulness and acceptance: A literature review. Behav Modif. 39, 870–911. doi: 10.1177/0145445515603707 26349756

[ref42] Lustig C. Hasher L. Zacks R. T. (2007). Inhibitory deficit theory: recent developments in a “new view.”. Inhibition cog. 145–162. doi: 10.1037/11587-008

[ref43] Lutz A. Jha A. P. Dunne J. D. Saron C. D. (2015). Investigating the phenomenological matrix of mindfulness-related practices from a neurocognitive perspective. Am. Psychol. 70, 632–658. doi: 10.1037/a0039585, PMID: 26436313 PMC4608430

[ref44] Lutz A. Slagter H. A. Dunne J. D. Davidson R. J. (2008). Attention regulation and monitoring in meditation. Trends Cogn. Sci. 12, 163–169. doi: 10.1016/j.tics.2008.01.005, PMID: 18329323 PMC2693206

[ref45] Luu K. Hall P. A. (2017). Examining the acute effects of hatha yoga and mindfulness meditation on executive function and mood. Mindfulness 8, 873–880. doi: 10.1007/s12671-016-0661-2

[ref46] Lymeus F. Lindberg P. Hartig T. (2018). Building mindfulness bottom-up: meditation in natural settings supports open monitoring and attention restoration. Conscious. Cogn. 59, 40–56. doi: 10.1016/j.concog.2018.01.008, PMID: 29438869

[ref47] Malinowski P. (2013). Neural mechanisms of attentional control in mindfulness meditation. Front. Neurosci. 7:8. doi: 10.3389/fnins.2013.00008, PMID: 23382709 PMC3563089

[ref48] Meeûs M. S. P. De Cuyper B. Boen F. (2010). The effect of mindfulness training on performance in closed-skill sports: the power of mild acceptance. J. Sport Exerc. Psychol. 32, 199–200.

[ref49] Mendelson D. N. Redfern M. S. Nebes R. D. Richard Jennings J. (2010). Inhibitory processes relate differently to balance/reaction time dual tasks in young and older adults. Aging Neuropsychol. Cogn. 17, 1–18. doi: 10.1080/13825580902914040, PMID: 19526388 PMC4904295

[ref50] Monno A. Chardenon A. Temprado J. J. Zanone P. G. Laurent M. (2000). Effects of attention on phase transitions between bimanual coordination patterns: a behavioral and cost analysis in humans. Neurosci. Lett. 283, 93–96. doi: 10.1016/S0304-3940(00)00924-1, PMID: 10739883

[ref51] Monno A. Temprado J. J. Zanone P. G. Laurent M. (2002). The interplay of attention and bimanual coordination dynamics. Acta Psychol. 110, 187–211. doi: 10.1016/S0001-6918(02)00033-1, PMID: 12102105

[ref52] Naranjo J. R. Schmidt S. (2012). Is it me or not me? Modulation of perceptual-motor awareness and visuomotor performance by mindfulness meditation. BMC Neurosci. 13:88. doi: 10.1186/1471-2202-13-88, PMID: 22846109 PMC3523966

[ref53] Nassauer K. W. Halperin J. M. (2003). Dissociation of perceptual and motor inhibition processes through the use of novel computerized conflict tasks. J. Int. Neuropsychol. Soc. 9, 25–30. doi: 10.1017/S1355617703910034, PMID: 12570355

[ref54] Polak E. L. (2009). Impact of two sessions of mindfulness training on attention Open Access Diss. 251

[ref55] Prätzlich M. Kossowsky J. Gaab J. Krummenacher P. (2016). Impact of short-term meditation and expectation on executive brain functions. Behav. Brain Res. 297, 268–276. doi: 10.1016/j.bbr.2015.10.012, PMID: 26462570

[ref56] Prinz W. A. Koch I. (2009). “Cognition and action” in Oxford handbook of human action. eds. Morsella P. M. G. E. Bargh J. A. (United Kingdom: Oxford University Press), 33–77.

[ref57] Redfern M. S. Chambers A. J. Sparto P. J. Furman J. M. Jennings J. R. (2018). Perceptual inhibition associated with sensory integration for balance in older adults. Dement. Geriatr. Cogn. Disord. 46, 266–274. doi: 10.1159/000493748, PMID: 30404094

[ref58] Redfern M. S. Chambers A. J. Sparto P. J. Furman J. M. Jennings J. R. (2019). Inhibition and decision-processing speed are associated with performance on dynamic posturography in older adults. Exp. Brain Res. 237, 37–45. doi: 10.1007/s00221-018-5394-0, PMID: 30302490 PMC6438625

[ref59] Redfern M. S. Jennings J. R. Mendelson D. Nebes R. D. (2009). Perceptual inhibition is associated with sensory integration in standing postural control among older adults. J. Gerontol. B. Psychol. Sci. Soc. Sci. 64, 569–576. doi: 10.1093/geronb/gbp060, PMID: 19617457 PMC2800814

[ref60] Rigoli D. Piek J. P. Kane R. Oosterlaan J. (2012). Motor coordination, working memory, and academic achievement in a normative adolescent sample: testing a mediation model. Arch. Clin. Neuropsychol. 27, 766–780. doi: 10.1093/arclin/acs061, PMID: 22777140

[ref61] Robertson D. G. E. Dowling J. J. (2003). Design and responses of Butterworth and critically damped digital filters. J. Electromyogr. Kinesiol. 13, 569–573. doi: 10.1016/S1050-6411(03)00080-4, PMID: 14573371

[ref62] Salter J. E. Wishart L. R. Lee T. D. Simon D. (2004). Perceptual and motor contributions to bimanual coordination. Neurosci. Lett. 363, 102–107. doi: 10.1016/j.neulet.2004.03.071, PMID: 15172094

[ref63] Scholz J. P. Kelso J. A. S. (1990). Intentional switching between patterns of bimanual coordination depends on the intrinsic dynamics of the patterns. J. Mot. Behav. 22, 98–124. doi: 10.1080/00222895.1990.10735504, PMID: 15111283

[ref64] Schöner G. Kelso J. A. S. (1988). Dynamic pattern generation in behavioral and neural systems. Science 239, 1513–1520. doi: 10.1126/science.3281253 3281253

[ref65] Serrien D. J. Ivry R. B. Swinnen S. P. (2007). The missing link between action and cognition. Prog. Neurobiol. 82, 95–107. doi: 10.1016/j.pneurobio.2007.02.003 17399884

[ref66] Sleimen-Malkoun R. Devillers-Réolon L. Temprado J.-J. (2023). A single session of mindfulness meditation may acutely enhance cognitive performance regardless of meditation experience. PLoS One 18:e0282188. doi: 10.1371/JOURNAL.PONE.0282188, PMID: 36920902 PMC10016675

[ref67] Slimani M. Bragazzi N. L. Tod D. Dellal A. Hue O. Cheour F.. (2016). Do cognitive training strategies improve motor and positive psychological skills development in soccer players? Insights from a systematic review. J. Sports Sci. 34, 2338–2349. doi: 10.1080/02640414.2016.1254809, PMID: 27842463

[ref68] Smith-Ray R. L. Hughes S. L. Prohaska T. R. Little D. M. Jurivich D. A. Hedeker D. (2015). Impact of cognitive training on balance and gait in older adults. J. Gerontol. B. Psychol. Sci. Soc. Sci. 70, 357–366. doi: 10.1093/geronb/gbt097 24192586 PMC4542642

[ref69] Stroop J. R. (1935). Studies of interference in serial verbal reactions. J. Exp. Psychol. 18, 643–662. doi: 10.1037/h0054651

[ref70] Stuhr C. Hughes C. M. L. Stöckel T. (2018). Task-specific and variability-driven activation of cognitive control processes during motor performance. Sci. Rep. 8, 10811–10819. doi: 10.1038/s41598-018-29007-3, PMID: 30018399 PMC6050332

[ref001] Temprado J. J. Chardenon A. Laurent M. (2001). Interplay of biomechanical and neuromuscular constraints on pattern stability and attentional demands in a bimanual coordination task in human subjects. Neurosci. Lett. 303, 127–131. doi: 10.1016/S0304-3940(01)01650-0 11311509

[ref71] Temprado J. J. Monno A. Zanone P. G. Kelso J. A. S. (2002). Attentional demands reflect learning-induced alterations of bimanual coordination dynamics. Eur. J. Neurosci. 16, 1390–1394. doi: 10.1046/j.1460-9568.2002.02190.x, PMID: 12405998

[ref72] Temprado J. J. Swinnen S. P. Carson R. G. Tourment A. Laurent M. (2003). Interaction of directional, neuromuscular and egocentric constraints on the stability of preferred bimanual coordination patterns. Hum. Mov. Sci. 22, 339–363. doi: 10.1016/S0167-9457(03)00049-6, PMID: 12967762

[ref73] Temprado J.-J. Torre M. M. Langeard A. Julien-Vintrou M. Devillers-Réolon L. Sleimen-Malkoun R.. (2020). Intentional switching between bimanual coordination patterns in older adults: is it mediated by inhibition processes? Front. Aging Neurosci. 12:29. doi: 10.3389/fnagi.2020.00029, PMID: 32132919 PMC7041435

[ref74] Temprado J.-J. Vercruysse S. Salesse R. Berton E. (2010). A dynamic systems approach to the effects of aging on bimanual coordination. Gerontology 56, 335–344. doi: 10.1159/000262445, PMID: 19940462

[ref75] Temprado J. J. Zanone P. G. Monno A. Laurent M. (1999). Attentional load associated with performing and stabilizing preferred bimanual patterns. J. Exp. Psychol. Hum. Percept. Perform. 25, 1579–1594. doi: 10.1037/0096-1523.25.6.1579

[ref76] Wenk-Sormaz H. (2005). Meditation can reduce habitual responding. Altern. Ther. Health Med. 11, 42–58. PMID: 15819448

[ref77] Zanesco A. P. King B. G. MacLean K. A. Saron C. D. (2018). Cognitive aging and long-term maintenance of Attentional improvements following meditation training. J. Cogn. Enhanc. 2, 259–275. doi: 10.1007/s41465-018-0068-1

